# Reliable identification of mycobacterial species by PCR-restriction enzyme analysis (PRA)-*hsp65 *in a reference laboratory and elaboration of a sequence-based extended algorithm of PRA-*hsp65 *patterns

**DOI:** 10.1186/1471-2180-8-48

**Published:** 2008-03-20

**Authors:** Erica Chimara, Lucilaine Ferrazoli, Suely Yoko Misuka Ueky, Maria Conceição Martins, Alan Mitchel Durham, Robert D Arbeit, Sylvia Cardoso Leão

**Affiliations:** 1 Instituto Adolfo Lutz, São Paulo, Brazil; 2 Instituto de Matemática e Estatística, Universidade de São Paulo, São Paulo, Brazil; 3 Tufts University School of Medicine, Division of Geographic Medicine and Infectious Diseases, Boston, Massachusetts, USA; 4 Departamento de Microbiologia, Imunologia e Parasitologia, Universidade Federal de São Paulo, São Paulo, Brazil

## Abstract

**Background:**

Identification of nontuberculous mycobacteria (NTM) based on phenotypic tests is time-consuming, labor-intensive, expensive and often provides erroneous or inconclusive results. In the molecular method referred to as PRA-*hsp65*, a fragment of the *hsp65 *gene is amplified by PCR and then analyzed by restriction digest; this rapid approach offers the promise of accurate, cost-effective species identification. The aim of this study was to determine whether species identification of NTM using PRA-*hsp65 *is sufficiently reliable to serve as the routine methodology in a reference laboratory.

**Results:**

A total of 434 NTM isolates were obtained from 5019 cultures submitted to the Institute Adolpho Lutz, Sao Paulo Brazil, between January 2000 and January 2001. Species identification was performed for all isolates using conventional phenotypic methods and PRA-*hsp65*. For isolates for which these methods gave discordant results, definitive species identification was obtained by sequencing a 441 bp fragment of *hsp65*. Phenotypic evaluation and PRA-*hsp65 *were concordant for 321 (74%) isolates. These assignments were presumed to be correct. For the remaining 113 discordant isolates, definitive identification was based on sequencing a 441 bp fragment of *hsp65*. PRA-*hsp65 *identified 30 isolates with *hsp65 *alleles representing 13 previously unreported PRA-*hsp65 *patterns. Overall, species identification by PRA-*hsp65 *was significantly more accurate than by phenotype methods (392 (90.3%) vs. 338 (77.9%), respectively; p < .0001, Fisher's test). Among the 333 isolates representing the most common pathogenic species, PRA-*hsp65 *provided an incorrect result for only 1.2%.

**Conclusion:**

PRA-*hsp65 *is a rapid and highly reliable method and deserves consideration by any clinical microbiology laboratory charged with performing species identification of NTM.

## Background

The genus *Mycobacterium *comprises organisms that are heterogeneous in terms of metabolism, growth, environmental niche, epidemiology, pathogenicity, geographic distribution and disease association [1]. While there are notable pathogens such as *Mycobacterium tuberculosis, Mycobacterium bovis *and *Mycobacterium leprae*, most are environmental organisms typically acting as opportunistic pathogens. These species, often collectively called nontuberculous mycobacteria (NTM), have been associated with a variety of problems including pulmonary, lymph node, skin, soft tissue, skeletal, and disseminated infections as well as nosocomial outbreaks related to inadequate disinfection/sterilization of medical devices [2]. In recent years, infections due to the subset of rapidly growing NTM, including *Mycobacterium fortuitum*, *Mycobacterium chelonae *and *Mycobacterium abscessus*, have been reported as complications of numerous surgical procedures, particularly involving foreign bodies (e.g., augmentation mammaplasty), high risk sites (e.g., eye) and injections of natural products used as alternative medicines [3-8].

In most laboratories, identification of mycobacterial species is based on in vitro growth and metabolic activities. Such phenotypic tests are labor-intensive and time-consuming to perform and may take several days to weeks to complete. Further, for many NTM species, the tests may be poorly reproducible [9], and consequently, the identifications may be ambiguous or erroneous [10].

DNA-based methods offer the promise of rapid and accurate species identification. However, commercially available DNA probes are available only for a handful of mycobacterial species; moreover, reagents are quite costly. Nucleotide sequence analyses can be used to resolve essentially any bacterial species, but requires both amplification and sequencing.

Telenti and coworkers described a DNA-based method for species identification of mycobacteria in which a portion of *hsp65*, the gene encoding the 65 kDa heat shock protein, was amplified by PCR and then analyzed by restriction digest [11]. This approach, referred to as PRA-*hsp65*, required only routine PCR and agarose gel electrophoresis equipment and could be completed within a few hours. The different species of mycobacteria yielded distinctly different patterns of restriction fragments and thus the species of an unknown isolate could be determined by comparing the fragments observed with published analyses of clinical isolates [11-17] and of newly described species [4,18-24]. The availability of an on-line internet resource facilitates the process [25].

Some studies have observed limitations to PRA-*hsp65 *which could, potentially, render the approach impractical for routine use. First, within commonly encountered species of clinical significance, such as *Mycobacterium avium *and *Mycobacterium kansasii*, as many as six distinct PRA-*hsp65 *patterns have been encountered [20,26-28]. Such variability could result in a high frequency of ambiguous or uninterpretable patterns. Second, validated protocols for electrophoresis and internal standards have not been defined [17,29]. Lastly, published tables present patterns which differ within a range of 5–15 bp and lack patterns for recently described species [11,14,16]. The aim of this study was to determine whether PRA-*hsp65 *of mycobacterial isolates provides sufficiently reliable species identification to enable it to be used as the routine methodology in a reference laboratory.

## Results

### Species identification by phenotype and PRA-*hsp65 *considered separately

Among the 434 isolates studied, biochemical and phenotypic evaluation alone assigned 371 (85.5%) isolates a species or complex; PRA-*hsp65 *assigned 404 (93%) isolates a species. Inconclusive results were obtained for 63 (14.5%) isolates by conventional methods compared with 30 (6.9%) isolates using the rapid DNA-based approach; these included nine isolates that could not be identified by either method.

### Species identification by phenotype and PRA-*hsp65 *compared to sequencing

For 321 (74.0%) of the 434 isolates both methods gave the same species identification, i.e., the results were concordant (Table 1). Based on prior experience by the authors and others [26,30], these identifications were presumed to be correct. The *hsp65 *genes of the remaining 113 (26.0%) isolates giving discordant or inconclusive results were sequenced. Among these, phenotypic testing had assigned 50 isolates to a species or a complex, but sequencing indicated that 33 (66%) of these assignments were incorrect (Table 2). For 63 isolates the phenotypic results were ambiguous and provided only a broad Runyon classification. Even among these, 19 (30.2%) were misclassified compared to conventional expectations [9,31], including 12 with regard to rate of growth (i.e., slow vs. rapid) and 7 with regard to chromogen production (Table 2). Overall, phenotypic species identification was correct for only 17 (15%) of 113 isolates for which *hsp65 *sequencing was performed.

**Table 1:** Species identification of 321 isolates which had concordant results by both phenotypic and PRA-*hsp65 *methods.

**Phenotypic identification**	**PRA-*hsp65*^b^**	**N (%)**
*M. avium *complex (146)^a^	*M. avium *1	107 (33.5)
	*M. avium *2	24 (7.5)
	*M. avium *3	1 (0.3)
	*M. intracellulare *1	13 (4.1)
	*M. intracellulare *4	1 (0.3)
*M. kansasii *(95)	*M. kansasii *1	95 (29.7)
*M. gordonae *(30)	*M. gordonae *1	2 (0.6)
	*M. gordonae *3	19 (6.0)
	*M. gordonae *4	2 (0.6)
	*M. gordonae *5	1 (0.3)
	*M. gordonae *7	3 (0.9)
	*M. gordonae *8	3 (0.9)
*M. fortuitum *complex (24)	*M. fortuitum *1	21 (6.6)
	*M. peregrinum *2	1 (0.3)
	*M. peregrinum *3	2 (0.6)
*M. chelonae *complex (21)	*M. chelonae *1	5 (1.6)
	*M. abscessus *1	14 (4.4)
	*M. abscessus *2	2 (0.6)
*M. marinum *(2)	*M. marinum *1	2 (0.6)
*M. terrae *complex (2)	*M. terrae *1	1 (0.3)
	*M. nonchromogenicum *2	1 (0.3)
*M. szulgai *(1)	*M. szulgai *1	1 (0.3)

^a ^Number of isolates.

^b ^PRA-*hsp65 *designation; see text for details.

**Table 2:** Results for 96 NTM isolates for which phenotypic methods gave incorrect species identification as determined by *hsp65 *sequencing.

**Species**	**N^b^**	**Phenotypic result**
*M. abscessus *(1)^c^	1	SGN
*M. arupense *(5)	1	*M. chelonae *complex
	2	SGN
	1	SGS
*M. asiaticum *(3)	2	*M. avium *complex
	1	*M. gordonae*
*M. avium *(18)	1	*M. chelonae *complex
	1	*M. fortuitum*
	2	*M. kansasii*
	10	SGN
	1	RGN
	1	SGP
	1	SGS
*M. celatum *(2)	1	*M. xenopi*
	1	SGN
*M. chelonae *(2)	2	SGN
*M. cosmeticum *(1)	1	*M. chelonae*
*M. farcinogenes *(1)	1	*M. chelonae *complex
*M. flavescens *(1)	1	RGS
*M. fortuitum *(6)	2	*M. chelonae *complex
	1	RGN
	1	SGN
*M. genavense *(1)	1	SGN
*M. gordonae *(26)	1	RGP
	1	SGN
	12	SGS
*M. hassiacum *(1)	1	RGS
*M. intracellulare *(9)	1	*M. chelonae *complex,
	1	*M. gordonae*
	7	SGN
*M. kansasii *(7)	1	*M. nonchromogenicum*
	2	RGP
	1	SGN
	2	SGP
	1	SGS
*M. lentiflavum *(3)	2	*M. avium *complex
	1	*M. gordonae*
*M. mageritense *(1)	1	*M. fortuitum*
*M. marinum *(1)	1	*M. kansasii*
*M. mucogenicum *(8)	2	*M. chelonae *complex
	1	*M. fortuitum *complex
	1	*M. peregrinum*
	3	SGN
	1	SGS
*M. nebraskense *(1)	1	*M. gordonae*
*M. nonchromogenicum *(2)	1	SGN
*M. peregrinum *(4)	4	*M. chelonae *complex
*M. phlei *(1)	1	RGS
*M. scrofulaceum *(3)	1	*M. avium *complex
	2	SGN
*M. sherrisii *(3)	2	*M. avium *complex
	1	SGN
*M. szulgai *(1)	1	SGS
*M. terrae *(2)	1	SGN

^a ^Species identification was determined by *hsp65 *sequencing for 113 isolates that had discordant results by PRA-*hsp65 *and phenotypic studies. For 17 isolates sequencing confirmed the species identification obtained by phenotypic methods.

^b ^Number of isolates for which the phenotypic identification shown was incorrect.

^c ^Total number of isolates of that species sequenced. SGS: slowly growing scotochromogen; SGN: slowly growing nonchromogen; SGP: slowly growing photochromogen; RGS: rapidly growing scotochromogen; RGN: rapidly growing nonchromogen; RGP: rapidly growing photochromogen.

Among the 113 isolates with discordant or inconclusive results, PRA-*hsp65 *assigned 83 isolates to a species; 71 (85.5%) of these assignments were confirmed by *hsp65 *partial gene sequencing (Table 3). For most of the remaining isolates, the identifications resolved by PRA-*hsp65 *and sequencing were consistent with close evolutionary relationships (e.g., *M. kansasii *and *Mycobacterium gastri*, *Mycobacterium intracellulare *and *M. avium) *(Table 3).

**Table 3:** Results for 12 NTM isolates for which PRA-*hsp65 *gave incorrect species identification as determined by *hsp65 *sequencing.

**Species**	**N^b^**	**PRA-*hsp65 *result**
*M. avium *(18)^c^	1	*M. kansasii *1
*M. farcinogenes *(1)	1	*M. scrofulaceum *1
*M. intracellulare *(9)	1	*M. avium *3
*M. kansasii *(7)	1	*M. avium *2
	1	*M. gastri *1
*M. mucogenicum *(8)	1	*M. chitae *1
	1	*M. gordonae *1
	1	*M. nonchromogenicum *1
*M. nebraskense *(1)	1	*M. avium *3
*M. scrofulaceum *(3)	2	*M. lentiflavum *3
	1	*M. simiae *1

^a ^Species identification was determined by *hsp65 *sequencing for 113 isolates that had discordant results by PRA-*hsp65 *and phenotypic studies. For 71 isolates sequencing confirmed the species identification obtained by PRA-*hsp65*. For an additional 30 isolates, the PRA-*hsp65 *patterns obtained were previously unreported (see Table 4).

^b ^N, number of isolates for which the PRA-*hsp65 *identification shown was incorrect.

^c ^Total number of isolates of that species sequenced.

There were 30 isolates representing 13 PRA-*hsp65 *patterns not in the available databases and the species was resolved by sequencing. The observed BstEII and HaeIII fragments for these new patterns (designated NP), the source of these isolates and the species identification based on sequencing are listed in Table 4; the observed phenotypes, including antimicrobial susceptibilities, are presented in Table 5. In four instances (NP1, NP11, NP14 and NP17, representing *Mycobacterium gordonae Mycobacterium terrae, Mycobacterium sherrisii *and *Mycobacterium arupense*, respectively) multiple isolates with the pattern were identified.

**Table 4:** BstEII and HaeIII fragment lengths (base pairs) for 30 isolates with new patterns by PRA-*hsp65*.

**Species^a^**	**PRA-*hsp65***	**N**	**Fragment BstEII**	**Length (bp) HaeIII**
***M. arupense***	**NP17**	**5**	**320-115**	**145-75-60**
*M. avium*	NP10	1	320-115	140-90-60
*M. cosmeticum*	NP6	1	320-115	150-95-80
*M. fortuitum*	NP12	1	235-120-85	140-120-100-55
*M. fortuitum*	NP19	1	235-120-100	145-140-100-55
***M. gordonae***	**NP1**	**11**	**235-120-100**	**130-110-95**
*M. gordonae*	NP3	1	320-130	130-60
*M. gordonae*	NP13	1	235-120-85	130-90
*M. gordonae*	NP22	1	235-130-85	160-90-60
*M. mageritense*	NP5	1	240-130-85	145-100-50
*M. nonchromogenicum*	NP4	1	235-120-85	145-80-60
***M. sherrisii***	**NP14**	**3**	**235-120-85**	**145-130**
***M. terrae***	**NP11**	**2**	**235-210**	**140-115-70**

^a ^Species identification based on sequencing of *hsp65 *gene. Bold indicates sequences submitted to GenBank and patterns included in the updated PRA-*hsp65 *algorithm (see Figures 1, 2 and 3). GenBank accession numbers: NP1, EF601222; NP11, EF601223; NP14, AY365190 [23]; NP17, DQ168662 [18].

All isolates with new PRA-*hsp65 *profiles were cultured from sputum, with the following exceptions: NP1: urine (2), feces, liver biopsy and unknown (one each); NP17: unknown (2).

**Table 5:** Phenotypic characteristics of isolates demonstrating previously unreported PRA-*hsp65 *patterns.

**Species**	**PRA *hsp65***	**25°C**	**37°C**	**45°C**	**pg**	**TCH**	**nit**	**Tween**	**NaCl**	**Aryl3**	**Aryl15**	**ag**	**pic**	**β-gal**	**LJ**	**HA**	**PNB**	**INH**	**RF**	**EMB**	**CIP**	**OFL**
*M. arupense*	NP17	3	3	0	N	2–3	0	1	0	0–1	0–2	nd	0	0–1	nd	3	2–3	3	0	0	0–1	3
*M. avium*	NP10	3	3	0	N	3	3	1	1	2	3	3	0	0	3	3	1	1	1	0	0	0
*M. cosmeticum*	NP6	3	3	2	N	3	0	1	0	1	2	1	2	1	nd	2	3	2	3	2	1	2
*M. fortuitum*	NP12	3	3	0	S	3	3	1	3	1	3	2	1	0	3	3	3	3	3	3	0	3
*M. fortuitum*	NP19	3	3	0	N	3	3	0	3	3	3	3	3	1	3	3	3	3	3	3	0	2
*M. gordonae*	NP1	2–3	3	0	S	3	0	1	0	1	2	0	0	0	3	1–3	3	0–3	0–3	0–1	0–1	1–3
*M. gordonae*	NP3	3	1	0	S	3	0	2	0	0	2	0	0	0	3	1	2	0	0	0	0	0
*M. gordonae*	NP13	1	2	0	P	3	1	0	nd	0	1	0	0	0	3	3	3	0	1	1	2	1
*M. gordonae*	NP22	3	3	3	S	3	3	2	0	0	0	nd	0	0	3	0	3	1	3	3	0	nd
*M. mageritense*	NP5	3	3	0	N	3	2	1	0	0	2	3	0	0	3	3	3	0	3	0	0	0
*M. nonchromogenicum*	NP4	2	3	2	N	2	1	1	0	0	0	nd	1	1	nd	2	2	2	2	0	0	0
*M. sherrisii*	NP14	1	2	0	S	1–2	0	0	1–2	0	0	1	0	1	2	1–2	1–2	1	1	2	2	1–2
*M. terrae*	NP11	2	3	0	N/S	2–3	0–3	1, 2	1	0	0–1	0	0	0–1	3	3	1, 2	3	0	0	0, 1	3

Phenotypes: **24°C, 36°C, 45°C**: growth at temperature shown; **pg**: pigmentation (N, nonchromogen; P, photochromogen; S, scotochromogen); **TCH**: growth on thiophene-2-carboxylic acid hydrazide; **nit**: nitrate reduction; **Tween**: hydrolysis of Tween 80; **NaCl**: growth on 5% NaCl; **Aryl 3. Aryl 15**: arylsulfatase activity after 3 and 15 days of growth, respectively; **ag**: growth on nutrient agar; **pic**: growth on picric acid; **β-gal**: β-galactosidase activity; **LJ**: growth on Löwenstein-Jensen media; **HA**: growth on hydroxylamine 500 μg/ml; **PNB**: growth on p-nitrobenzoic acid; **INH**: isoniazid; **RF**: rifampicin; **EMB**: ethambutol; **CIP**: ciprofloxacin; **OFL**: ofloxacin. Responses are graded 0 (negative, no growth, no activity expressed) to 3 (positive, heavy growth, strong activity expressed); nd, not done. For patterns with multiple isolates, the result shown represents the most common phenotype(s) or the range of phenotypes observed.

### Overview of results

The overall results of the two methods are summarized in Table 6. Among 434 NTM isolates, PRA-*hsp65 *provided correct species identification significantly more frequently than phenotypic/biochemical testing (392 (90.3%) vs 338 (77.9%), respectively; p < .0001, Fisher's exact test).

**Table 6:** Summary of concordance among species identification results obtained by PRA-*hsp65*, phenotypic evaluation and sequence analysis of the *hsp65 *gene.

***hsp65 *sequence**	**N**	**PRA-*hsp65***			**Phenotypic identification**		
			
		**Concordant**	**New Pattern**	**Discordant**	**Concordant**	**Ambiguous**	**Discordant**
Not done^a^	321	321	--	--	321	--	--
Done	113	71	30	12	17	63	33
Total	434	392 (90.3%)	30 (6.9%)	12 (2.8%)	338 (77.9%)	63 (14.5%)	33 (7.6%)

^a ^Isolates for which species identification by PRA-*hsp65 *and phenotypic/biochemical evaluation were concordant were not sequenced. Based on prior reports by the authors and others, sequencing *hsp65 *in such isolates almost invariably confirms the species identification of the other methods.

The four species or complex of NTM most commonly associated with clinically significant disease are *M. avium *complex, *M. fortuitum *complex, *M. chelonae *complex and *M. kansasii*. These represented 333 (76.7%) of the 434 isolates in this collection. PRA-*hsp65 *provided incorrect species identification for only 4 (1.2%) of these isolates and a new pattern for an additional 3 (0.9%). In contrast, phenotypic/biochemical testing provided incorrect assignments for 9 (2.7%) and ambiguous results for 31 (9.3%). Thus, the frequency of incorrect or uncertain species identification among these isolates of potential clinical importance was almost 6-fold higher for the phenotypic method than for PRA-*hsp65 *(40 (12.0%) vs. 7 (2.1%), respectively; p < .0001, Fisher's exact test).

### PRA-*hsp65 *algorithm

**Figure 1:**
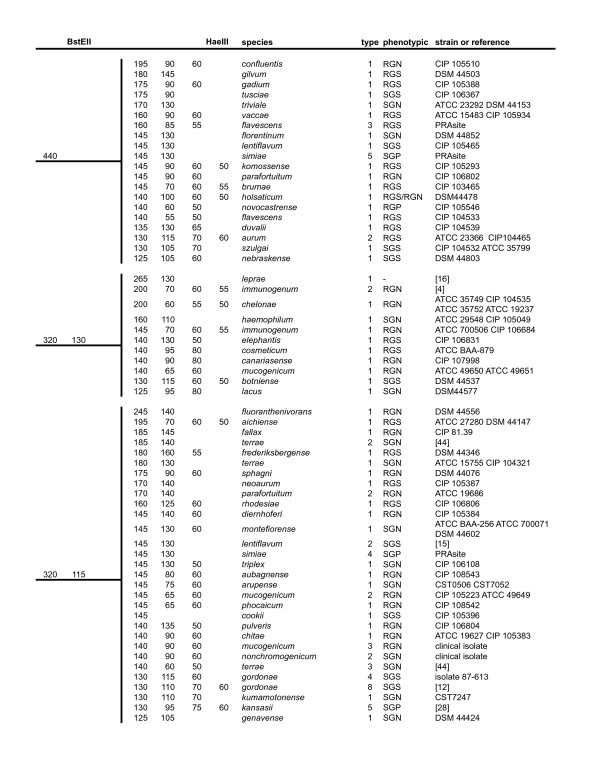
**Algorithm of PRA-*hsp65 *patterns based on analysis of the 441 bp fragment of the *hsp65 *gene. BstEII patterns: 440 bp, 320 bp/130 bp, 320 bp/115 bp**. Columns 1 and 2: calculated BstEII and HaeIII fragment sizes in base pairs. Column 3: species names according to [32]. Column 4: PRA-*hsp65 *pattern type. Column 5: RGN: rapidly growing non-pigmented, RGS: rapidly growing scotochromogen, RGP: rapidly growing photochromogen, SGN: slowly growing non-pigmented, SGS: slowly growing scotochromogen, SGP: slowly growing photochromogen. Column 6: strain(s) used for *hsp65 *sequencing or reference of the publication describing this pattern.

**Figure 2:**
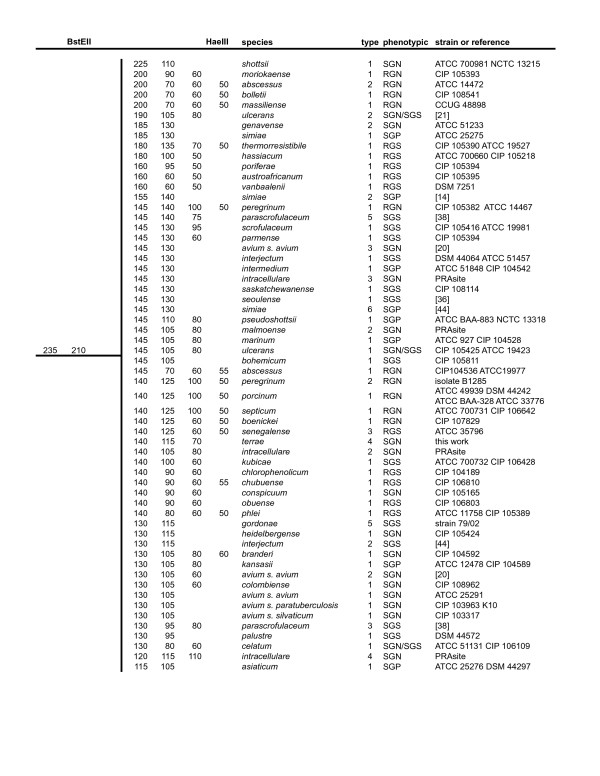
**Algorithm of PRA-hsp65 patterns based on analysis of the 441 bp fragment of the hsp65 gene. BstEII patterns: 235 bp/210 bp**. Columns 1 and 2: calculated BstEII and HaeIII fragment sizes in base pairs. Column 3: species names according to [32]. Column 4: PRA-*hsp65 *pattern type. Column 5: RGN: rapidly growing non-pigmented, RGS: rapidly growing scotochromogen, RGP: rapidly growing photochromogen, SGN: slowly growing non-pigmented, SGS: slowly growing scotochromogen, SGP: slowly growing photochromogen. Column 6: strain(s) used for *hsp65 *sequencing or reference of the publication describing this pattern.

**Figure 3:**
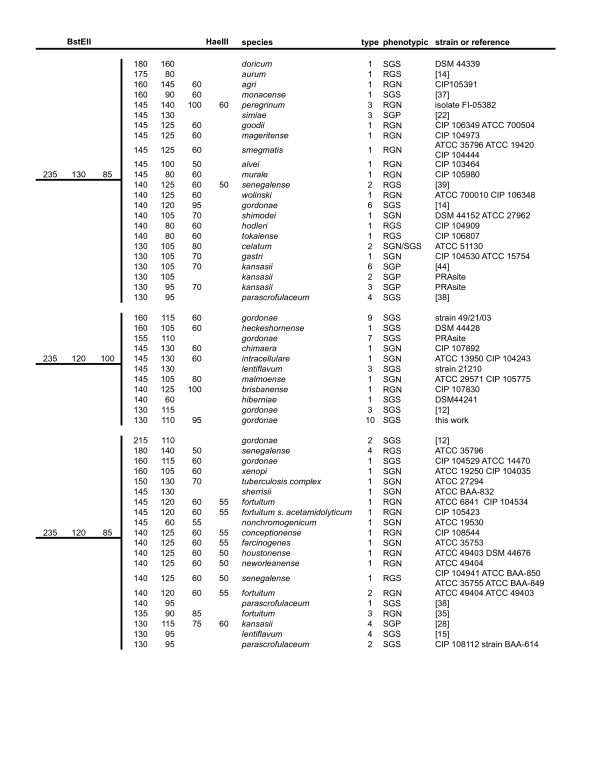
**Algorithm of PRA-*hsp65 *patterns based on analysis of the 441 bp fragment of the *hsp65 *gene. BstEII patterns: 235 bp/130 bp/85 bp, 235 bp/120 bp/100 bp, 235 bp/120 bp/85 bp**. Columns 1 and 2: calculated BstEII and HaeIII fragment sizes in base pairs. Column 3: species names according to [32]. Column 4: PRA-*hsp65 *pattern type. Column 5: RGN: rapidly growing non-pigmented, RGS: rapidly growing scotochromogen, RGP: rapidly growing photochromogen, SGN: slowly growing non-pigmented, SGS: slowly growing scotochromogen, SGP: slowly growing photochromogen. Column 6: strain(s) used for *hsp65 *sequencing or reference of the publication describing this pattern.

Figures 1, 2 and 3 display an updated algorithm relating observed restriction fragments to particular species. We have included refinements of previously assigned fragment sizes based on our observations and analysis of available *hsp65 *sequences from validated mycobacterial species found online [32]. Sequences retrieved from GenBank [33] comprising the 441 bp Telenti fragment were analyzed using BioEdit, version 7.0.5.3. [34] and/or the DNASIS Max version 1 program (Hitashi Software Engineering Co., USA). BstEII restriction patterns were distributed in seven possible configurations: 440, 320-130, 320-120, 235-210, 235-130-85, 235-120-100, and 235-120-85. HaeIII fragment sizes were adjusted considering the nearest number multiple of 5, to facilitate interpretation of gel bands. These adjustments were performed based in our experience with analysis of more than 500 gels both visually and using the GelCompar program. HaeIII restriction fragments shorter than 50 bp were not taken in account as their discrimination in 4% agarose gels is often inaccurate. Different variants of PRA-*hsp65 *profiles from each species were numbered using Arabic numbers after the designation of the species, as reported in the PRASITE, except for *M. avium*, for which variants *M. avium *1 and *M. avium *2 were defined as reported in Leao et al. [20] and Smole et al. [27]. There were also PRA-*hsp65 *patterns frequently found in our routine work that had no sequence deposited. These patterns were included according to published data [11-17] or the PRASITE [25]. Figures 2 and 3 also include the two new patterns we observed in two or more isolates (NP11 and NP1) and for which we propose PRA-*hsp65 *designations, *M. terrae *4 and *M. gordonae *10, respectively. The partial *hsp65 *gene sequences of these isolates have been deposited in GenBank [GenBank:EF601223 and GenBank:EF601222, respectively]. The figures also indicate the basic phenotypic characteristics (time for growth and pigment production) observed for each species.

## Discussion

The incidence of individual infections and outbreaks associated with NTM has risen dramatically over the past decade establishing these organisms as significant human pathogens. Traditionally, the identification of mycobacteria to the species level has relied upon biochemical tests, which require three to six weeks to complete. Biochemical identification, even when performed by skilled microbiologists, may yield uncertain or even misleading results because (a) the tests used are inherently poorly reproducible; (b) the expected phenotypes are not an absolute property of the species, but may exhibit substantial variability; and (c) the database of phenotypic characteristics is limited to common species [10].

In recent years, DNA-based techniques have greatly facilitated identifying the species of NTM isolates and enabled a number of new species to be documented as infecting agents [35-39]. These approaches can be applied to a single isolated colony and a definitive result can typically be obtained within a day. PRA-*hsp65*, first described by Telenti et al., is based on detection of restriction fragment polymorphisms in the *hsp65 *gene and thereby resolving the species of a mycobacterial isolate [11].

In the present study, 434 NTM isolates from clinical specimens were analyzed by conventional phenotypic methods and by PRA-*hsp65*; further, those isolates for which the results from the two methods were discordant were analyzed using nucleotide sequencing of the *hsp65 *gene. For 63 (14.5%) isolates phenotypic methods could not provide a species identification and for almost a third of these isolates even the apparent Runyon classification proved inconsistent with conventional expectations. For an additional 33 (7.6%) isolates the phenotypic identification proved incorrect. Phenotypic variability among fresh clinical isolates has been observed in other studies [10,40,41].

In contrast, PRA-*hsp65 *correctly identified over 90% of evaluable isolates using currently available databases of restriction digest patterns. For most of the remaining isolates, the PRA-*hsp65 *pattern observed was not previously reported. There were only 4 (1.2%) clinically significant isolates for which the current PRA algorithm indicated an incorrect species.

PRA-*hsp65 *has proven similarly effective in other studies. Hafner et al. used 16S rDNA sequencing to analyze 126 isolates selected at random from a larger collection [17]. The *hsp65 *method correctly identified 120 (95.2%) of these isolates. They also sequenced 10 additional isolates from the larger collection that gave PRA-*hsp65 *patterns not previously reported. All these isolates represented environmental species rarely associated with clinically significant disease.

Among our 434 isolates, 30 (6.9%) provided 13 PRA-*hsp65 *profiles not previously reported. Our series represents isolates cultured from varied clinical specimens collected in the metropolitan and surrounding areas of the city of Sao Paulo, Brazil. Most of the isolates with new PRA-*hsp65 *patterns were cultured from sputum. Many represented species typically considered non-pathogens; clinical correlation was not available and these isolates may reflect colonization by environmental organisms. Previous studies have similarly documented considerable species diversity as well as the genotypic diversity among mycobacteria isolates in Brazil [42,43]. Sequence analysis confirmed that the new profiles were allelic variations within the species, consistent with previous studies [13,17,20]. Of interest, four profiles were represented by more than one isolate, suggesting that they are potentially prevalent lineages rather than singular mutation events.

The most commonly identified new profile (designated NP1) was observed in 11 isolates, representing 20% of all *M. gordonae *in this collection. Comparison to the prototype *M. gordonae *sequence indicated two point mutations that resulted in the loss of two HaeIII sites and the addition of 95-bp fragment to the profile [GenBank:EF601222]. A similar profile was assigned to *M. gordonae *by da Silva Rocha et al. [13], although sequence confirmation was not reported. Hafner et al. also noted that *M. gordonae *is a particularly polymorphic species [17].

The NP17 profile, demonstrated for five isolates, was identified by sequencing as *M. arupense*, a recently described species related to the *M. terrae *complex [18]. The NP14 profile, observed for three *Mycobacterium simiae *isolates, was similar profile to the *M. simiae *3 pattern reported by Legrand et al. [22] as well as to the prototype *M. simiae *1 pattern [11]. Sequencing confirmed that the nucleotide sequence is intermediate between those two strains. The sequence also matches that recently reported by Selvarangan et al., who proposed that their isolates represented a new species (*M. sherrisii *sp. nov) based on a distinct pattern of cellular fatty acids and a unique 16S rRNA gene [23]. The NP11 profile, represented by two isolates of *M. terrae*, was similar to a PRA-*hsp65 *pattern described by McNabb et al. [44] with the addition of a unique HaeIII restriction site [GenBank:EF601223].

We would concur with Hafner et al. that additional work is required to define and standardize the most effective electrophoresis conditions for resolving *hsp65 *digests of mycobacteria [17]. In a recent multicenter study evaluating PRA-*hsp65*, variations related to gel preparation, running conditions and documentation tools all complicated the interpretation of digestion patterns [29].

The ever-increasing amount of data available and the identification of new profiles make the analysis more complex. We present an updated PRA-*hsp65 *algorithm, which includes 174 patterns among 120 species and sub-species and have the basic cultural characteristics (rate of growth and pigment production). These core phenotypic traits can be readily determined and, as emphasized in a recent statement by the American Thoracic Society [45], can assist in confirming the molecular identification, detecting mixed cultures, and classifying species with indistinguishable PRA-*hsp65 *patterns.

Despite the complexities noted above, PRA-*hsp65 *analysis proved both more rapid and more reliable than phenotypic methods; it was particularly effective at resolving the most common pathogenic species. Commercial DNA probes are available only for a very few species and their expense may be prohibitive in some settings. DNA sequencing is more definitive, but sequencing capability is not yet widely available in clinical laboratories.

## Conclusion

Based on our extensive practical experience, we believe that PRA-*hsp65 *has the potential to provide clinicians with more timely, more accurate and, ultimately, more useful information and therefore deserves consideration by any clinical microbiology laboratory charged with performing species identification on NTM.

## Methods

### Mycobacterial isolates

From January 2000 to January 2001, 5019 cultures were received at Institute Adolfo Lutz, São Paulo, Brazil for mycobacterial identification. *M. tuberculosis *complex was identified by direct observation of colony aspect and by Ziehl-Neelsen stained smears for presence/absence of cord formation. Cord-positive isolates with nonpigmented rough cultures were excluded from this study.

A total of 439 isolates consistent with NTM were cultured from 435 (8.7%) specimens; five isolates were excluded because they could not be unambiguously resolved as NTM by the three methods used (phenotypic, PRA-*hsp65 *and sequencing), leaving a total of 434 isolates in the study. The specimens yielding NTM included sputum (280), blood (41), bronchial lavage (13), bone marrow (13), urine (7), skin biopsy (6), lymph node (5), feces (6), corneal scraping (4), pleural fluid (4), ascitic fluid (2), liver biopsy (2), liquor (1), gastric fluid (1), synovial fluid (1), abscess/secretion from unknown origin (11) and unknown (38). The majority (61.4%) of these specimens were from the Metropolitan Region of São Paulo, with 36.1% from elsewhere in São Paulo State and 2.5% from other States in Brazil.

### Conventional identification

Isolates were identified based on phenotypic characteristics, including growth rate (fast/slow), pigment production, growth in different temperatures (26°C, 37°C and 45°C), biochemical tests (nitrate reduction, catalase activity, urease activity, tween 80 hydrolysis, arylsulfatase), specific chemicals (sodium chloride 5%, sodium salicylate), and growth in the presence of drugs (isoniazid 10 μg/ml, rifampicin 25 μg/ml, ethambutol 5 μg/ml, thiophen-2-carboxylic acid hydrazide 5 μg/ml, p-nitro-benzoic acid 0.5 μg/ml, cycloserine 30 μg/ml, ciprofloxacin 5 μg/ml, hydroxylamine 500 μg/ml, ofloxacin 2.5 μg/ml) [9,31]. Some closely related mycobacterial species cannot be resolved by these biochemical tests. In such instances, isolates were designated as *M. avium *complex, *M. terrae *complex, *M. chelonae *complex or *M. fortuitum *complex, as appropriate.

### DNA extraction and PRA-*hsp65 *method

For DNA extraction, a loop-full of organisms grown on Löwenstein-Jensen medium was suspended in 500 μl of ultrapure water, boiled for 10 min and frozen at -20°C for at least 18 h. Five microliters of DNA-containing supernatant were subjected to PCR amplification of the 441 bp of the gene *hsp65 *[11]. Separate aliquots of the PCR product were digested with BstEII and HaeIII, and the resulting restriction fragments separated by electrophoresis in a 4% agarose gel (Nusieve, FMC Bioproducts, Rockland, Maine USA) with 50 bp ladder as molecular size standard.

### Analysis of PRA-*hsp65 *results

Gels were stained with ethidium bromide, photographed on a UV transilluminator, the images scanned, the restriction fragment sizes estimated using GelCompar II software, version 2.5 (AppliedMaths, St. Marten Latem, Belgium) and the patterns observed compared to the patterns reported on PRASITE [25], in publications [11-17] or calculated *in silico *from sequences deposited in GenBank [33] using BioEdit, version 7.0.5.3 [34].

### *hsp65 *partial gene sequencing

For those isolates for which conventional and PRA-*hsp65 *methods gave discordant or inconclusive results, the *hsp65 *amplicon was purified using Novagen Spin-prep Kit (Novagen, Canada) and then sequenced using BigDye terminator cycle sequencing reagents. Cycle sequencing was performed by using a Perkin-Elmer 9600 GeneAmp PCR system programmed for 25 cycles at 96°C for 20 s, 50°C for 10 s and 60°C for 4 min. Sequencing products were cleaned with CentriSep Spin Columns (Princeton Separations, Applied Biosystems) and then analyzed on a ABI Prism 377 sequencer (Perkin-Elmer).

### Sequence data analysis

Data produced by the sequencer was automatically processed using the EGene platform [46]. The trace files were initially submitted to Phred [47] for base calling and quality assessment. Then, sequences were submitted to a quality filter that eliminated reads that did not present at least one window of 200 bases where 190 bases had phred quality above 15. After, low quality bases were trimmed from the sequence. For each sequence, the trimming procedure isolated a "good quality" subsequence. In this remaining subsequence, any window of 15 bases have at least 12 bases above the quality threshold of 15. After trimming, contaminant screening was performed using Blastn [48] against *Homo sapiens*, *Salmonella typhimurium *and *Gallus gallus *databases. Finally the clean isolates were identified by similarity using Blastn against a database of *hsp65 *genes. Sequences were considered a positive match when they presented a minimum similarity of 80 percent over a local alignment of at least 90 bases and ev-value of 1e-20. Species identification was confirmed if = 97% match was achieved, according to criteria proposed by McNabb et al. [44],. with any sequence deposited in databases and published.

## Authors' contributions

EC carried out the molecular genetic studies, participated in the sequence analysis and drafted the manuscript; LF participated in the design, initiation and coordination of the study; SYMU and MCM performed traditional identification studies; AMD participated in sequence analysis; RDA conceived the study and participated in its design and in the analysis of the results; SCL participated in the coordination of the study and in the analysis of results. All authors read and approved the final manuscript.
